# Barriers and motivators to participation and retention in HIV/HCV cohort studies among people who inject drugs: a community consultation in Iran

**DOI:** 10.1186/s13011-020-00298-y

**Published:** 2020-08-05

**Authors:** Ali Mirzazadeh, Samira Hosseini-Hooshyar, Armita Shahesmaeili, Ali Bahramnejad, Adibeh Barshan, Ghazal Mousavian, Esmail Najafi, Hamid Sharifi, Ali-Akbar Haghdoost, Alya Briceno, Willi McFarland, Kimberly Page

**Affiliations:** 1 HIV/STI Surveillance Research Center, and WHO Collaborating Center for HIV Surveillance, Institute for Futures Studies in Health, Kerman University of Medical Sciences, Kerman, Iran; 2 Department of Epidemiology and Biostatistics, Institute for Global Health Sciences, University of California San Francisco, 550 16th Street, San Francisco, CA 94158 USA; 3 The Kirby Institute, UNSW Sydney, Sydney, NSW Australia; 4 Kerman University of Medical Sciences, Kerman, Iran; 5 University of California San Francisco, San Francisco, CA USA; 6 Epidemiology, Biostatistics and Preventive Medicine, Department of Internal Medicine, University of New Mexico Health Sciences Center, Albuquerque, NM USA

**Keywords:** People who inject drugs, Longitudinal cohort study, Barriers, Motivators, Iran

## Abstract

**Background:**

The lack of robust estimates of HIV/HCV incidence among people who inject drugs (PWID) in Iran calls for well-designed prospective cohort studies. Successful recruitment and follow-up of PWID in cohort studies may require formative assessment of barriers PWID are faced with in participation and retention in cohort studies and factors they think may facilitate their engagement in such studies. Using a focus group discussion (FGD) format, we conducted a consultation with PWID in southeast Iran to recognize those barriers and motivators.

**Methods:**

Using targeted sampling and through snowball referrals, we recruited PWID (aged≥18, injected in last 6 months) from community-based drop-in centers (DICs), homeless shelters, and through outreach efforts to participate in four FGDs (one women-only). Socio-demographic characteristics, injection behaviors and self-reported HCV/HIV testing and diagnosis history were obtained. Then, a semi-structured FGD guide was applied to explore barriers and motivators to participation and retention in cohort studies among study participants. All FGD sessions were recorded and transcribed verbatim, removing any identifying information. The content of FGDs were analyzed by thematic analysis using an inductive approach.

**Results:**

In total, 30 individuals (10 women) participated in the study. The median age of participants was 35 (IQR 31–40), with majority (73.3%) reporting injecting drug use within the last month. Only 40.0% reported ever being tested for HCV whereas a larger proportion (63.4%) reported ever being tested for HIV. While the majority were willing to participate in cohort studies, breach of confidentiality, fear of positive test results, perceived required commitment, and marginalization were reported as barriers to participation and retention in such studies. Monetary incentive, the thought of a better life, protection from police interventions and trust between health workers and PWID were addressed as motivators of engagement in cohort studies among PWID.

**Conclusions:**

Strategies to enhance data security and reduce stigma associated with injecting drug use along with involving peer workers in research, providing pre and post-test counselling and education and addressing the needs of more marginalized groups potentially through integrated healthcare programs and housing support are among few approaches that may help address barriers and strengthen the motivators for successful cohort studies among this population.

## Background

Iran, straddling a major transit route of illicit drugs from Afghanistan to western countries [1], had the largest opiate seizures worldwide in 2014 [2]. Estimates suggest that there are around 208,000 people who inject drugs (PWID) currently living in Iran [3] who bear the highest burden of HIV [4, 5] and HCV [6, 7] among all at-risk populations. A recent systematic review indicated that there has not been any significant change in the prevalence of HIV before (14.3% [95% confidence interval (CI); 9.8–18.9]) and after the beginning of 2007 (9.7% [95% CI, 7.6–11.9]) among PWID in Iran [8]. The ongoing high-risk injection and sexual behaviors have been suggested to be correlated with this unchanged prevalence among this population [9]. Additionally, an estimated anti-HCV prevalence of 52.5% has been reported among Iranian PWID [7], a figure which is considered to be on the rise in the country [10].

Iran’s response to opiate dependence and its associated harms gradually shifted in the late 1990s from a supply-reduction policy criminalizing any type and quantity of drug use to implementing drug treatment and harm reduction programs across the country [11–13]. Current harm reduction programs catered towards PWID in Iran include education, access to needle and syringe program (NSP) and opioid substitution therapy (OST). Further, HIV testing and referral is often available through voluntary counselling and testing centers (VCTC), drop-in centers (DIC), and recently via established but limited number of mobile clinics [14]. The management of HCV, on the other hand, continues to occur in tertiary healthcare which has failed to provide access to HCV care among the more marginalized populations, including people with drug use disorders [15, 16]. Assessing and monitoring the impact of harm reduction interventions among PWID, require direct measures of incidence and predisposing factors of HIV, HCV and other common infections among this population [14, 17].

Very little evidence is available on directly measured HIV/HCV incidence among PWID in Iran. HCV epidemiological studies have been largely limited to single-centered cross-sectional studies reporting prevalence and with heterogeneous study populations with respect to their risk behaviors [18–24]. With regard to HIV incidence estimation, research has been limited to modelling studies [25] or not nationally representative studies [26]. Providing robust and reliable estimates of HIV/HCV incidence needs well-designed prospective cohort studies. Cohort studies of PWID are also critical to measure longitudinal changes in substance use disorders and risk behaviors. Intersections of drug use, mental health and blood-born infectious diseases and the effectiveness of harm reduction services is not well studied in most developing countries including Iran. HCV among PWID is mostly overlooked and is on raise in Iran [10]. Monitoring the overdose mortality and other cause mortality among PWID are other important health outcomes that need to be measured by cohort studies. Understanding who is at risk for such infections is the key to adapt prevention strategies to local settings and the population of interest. Iran produces highly effective generic sofosbuvir/daclatasvir/Ribavirin (with sustained virological response [SVR]; 92%) [27] for HCV genotype 1 and 3 (i.e. most prevalent HCV genotypes in Iranian people with HCV infection) [28]. However, studies to measure the coverage of HCV screening programs and access to generic HCV medications among PWID are lacking.

Designing and implementing prospective cohort studies are often challenging due to time and budget constraints in Iran [14]. Therefore, they need to be planned thoroughly to address all possible eventualities and exceptions. In addition, a successful recruitment and more importantly, long-term follow-up of PWID in prospective cohort studies requires formative assessment information [29] on challenges and obstacles that PWID are faced with in participation and retention in long-term studies and motivating factors and incentives that they think would help address those barriers and pave the way to their successful engagement in research. Therefore, in this qualitative study, using a focus group format, we aimed to better understand the perceptions, concerns, barriers and motivators to participation and retention in HIV/HCV cohort studies among PWID in an urban setting in Iran. The findings of this study has insightful implications for the direction of future research with the understudied population of PWID and perhaps other marginalized groups in Iran and similar settings.

## Methods

### Study design

Due to the dearth of data related to the subject matter of this research, community consultation [30] through focus groups discussion (FGD) was applied to generate detailed and in-depth information on participation and retention of PWID in HIV/HCV cohort studies. We preferred FGDs to one-on-one interviews (i.e. in-depth interviews) since we judged the former to be a more suitable approach to inspire brainstorming and allow people to generate ideas through exploring their shared knowledge and experience [31, 32]. FGDs also provided us the opportunity to identify reflections and expressions of participants towards one another [33].

### Study setting and participants

The study was conducted in a community-based DIC which is one of the four DICs serving PWID under the supervision of Kerman University of Medical Sciences in Kerman. Kerman is a city located in the south-east of Iran with a population of around 738,000 [34]. Community-based DICs provide free-of-charge services including sterile needle and syringes for injection, condoms, warm meals and personal hygiene facilities [35] for at-risk populations. Some additional services are often provided in Kerman DICs including OST, HIV screening and sexual health education and some other social services.

Participants were recruited using targeted sampling [36] that integrated various efforts including snowball method (i.e. chain referrals), word of mouth, talking to people in DICs, calling homeless shelters, and recruitment through outreach efforts. Targeted sampling was adopted as the method of choice in order to reach those individuals who may not be connected to services or associated with networks such as homeless individuals [37]. Outreach workers were locals who were familiar with PWID community and collaborated with OST clinics or NSPs to help find and link PWID to harm reduction services.

With the help of peer workers (former PWIDs), we attempted to include people from various settings, with different patterns and types of drug use/injection, different accessibility to services and different HIV serostatus to ensure a diverse participant pool with respect to characteristics and perspectives on barriers and facilitators of engaging in HIV/HCV research. In addition, a women-only group was recruited to further explore gender differences with regard to the topic of this study.

Eligibility criteria for all participants included age ≥ 18 years, speaking Farsi (i.e. Persian) as the first language, residing or working in Kerman for the past 6 months and reporting injecting any type of illicit drug for non-medical purposes in the last 6 months. Research assistants looked for any recent injection marks to confirm the injection status of participants.

### Study procedures

Upon arrival to the DIC, the eligibility was verified and written informed consent was obtained for each participant. Participants also completed a short interviewer-administered survey in a private room to provide socio-demographic characteristics (i.e. age, sex, education, ethnicity and current living status), injection behaviors (i.e. age at first injection, drug injection in the last month, frequency of injection in the last month, any drugs used and injected in the last 6 months and last 1 month, and main drugs used and injected in last 6 months), and self-reported HCV and HIV testing and diagnosis history (i.e. HCV and HIV testing, HCV and HIV serostatus and HCV RNA status).

A semi-structured FGD guide with open-ended questions as well as introductory questions (Supplemental Appendix 1) was applied to explore barriers and motivators to participation and retention in HIV/HCV cohort studies among study participants. An HIV/HCV cohort study was defined for participants as a long-term prospective study of PWID to evaluate the incidence of HIV infection and HCV (re) infection and referring people to treatment programs upon diagnosis of either HIV or HCV infection. The guide was applied to familiarize, direct, and focus the participants on the purpose of the study and probed four topics including 1) participation in HIV/HCV cohort studies; 2) retention in such studies; 3) inviting and engaging peers and partners (i.e. sex and injecting partners) in such studies; and 4) logistics needed (i.e. preferred time, location and other circumstances for attending the study screening and follow-ups) for such studies.

A series of four FGDs (one women-only) were carried out at the designated DIC from April 26 to May 15, 2016. Each focus group was moderated by an experienced qualitative researcher. Two other members of the research team served as nonparticipant observers/note-takers who audio-recorded and documented observations during FGDs. The group moderator and observers were gender-matched for each group. To facilitate participation in each FGD, the moderator started by assuring participants that there is no right or wrong answer and asked them to introduce themselves by their pseudonyms and share something they would like the group to know about them. The moderator also ensured that the group did not distract from the topic of discussion and that no member dominated the discussion.

FGDs lasted up to 90 min and took place in a room with a neutral and peaceful environment at DIC that allowed for privacy and comfort of participants. Audio-recordings were transcribed verbatim, removing any identifying information. Written transcripts were reviewed while listening to the original audio-recordings, compared with notes taken by note takers to fill in any missing words and erased from recording equipment. Upon the conclusion of each FGD, participants received a small monetary incentive (equivalent to 4 USD) in return for their participation.

### Data analysis

The survey data and verbatim transcripts were the primary data for analysis in this study. Descriptive statistics (i.e. median and interquartile range (IQR) for continuous variables, and frequency and percentages for categorical variables) were applied in STATA v 13.1 (StataCorp, College Station, Texas) to summarize participants characteristics.

For the analysis of transcripts, two investigators conducted an inductive thematic analysis [38]. No analytical software was used. In the first step, investigators independently reviewed all transcripts for main themes and subcategories. They then developed consensus over a set of preliminary codes and coded the transcripts line by line. Refining and creating additional codes were carried out and discussed when necessary. Discrepancies were resolved by discussion to reach consensus. Finally, codes were grouped into categories.

## Results

In total, 20 men and 10 women participated in the study (Table 1), no one refused to participate in the study. The four focus groups ranged in size from 5 to 12 participants, with the vast majority having a Farsi ethnicity (*n* = 29) and only one person being Azari (Turk). The median age of participants was 35 years (IQR 31–40), the majority had either a primary (23.3%, *n* = 7) or middle (33.3%, *n* = 10) school level of education. Most participants were currently living in shelters (30.0%, *n* = 9), seven individuals (23.3%) had their own house to live in, five (16.7%) were currently living with friends, family or partner and three (10.0%) were homeless. Nearly half of participants (43.3%, *n* = 13) were currently living with a same-sex friend, eight (26.7%) were living alone and six (20.0%) were currently living with their spouse and children. The median age at first injecting was 23.5 years (IQR 19–30) among participants and 73.3% (*n* = 22) reported injecting drug use within the last month. Among those reporting injecting drug use in the last month, the median number of days injected drugs in the last month was 5 (range 1, 30) and the median number of injections per day in the last month was 1 (range 1, 3).

**Table 1:** Characteristics of participants in focus group discussions of barriers and motivators to participation and retention in cohort studies in Iran

Variables	***N*** = 30
Age (years), median (IQR)	35 (31–40)
Sex, n (%)	
Female	10 (33.3)
Male	20 (66.7)
Education, n (%)	
Illiterate/Read and write only	3 (10.0)
Primary school	7 (23.3)
Middle school	10 (33.3)
High school	4 (13.3)
Diploma or higher	6 (20.1)
Ethnicity, n (%)	
Fars	29 (96.7)
Azari/Turk	1 (3.3)
Current living place, n (%)	
Own house	7 (23.3)
Parents’ house	2 (6.7)
Friends, family or partner house	5 (16.7)
Shelter	9 (30.0)
Homeless	3 (10.0)
Others (not specified)	4 (13.3)
Currently living with, n (%)	
Spouse and children	6 (20.0)
Any partner	1 (3.3)
Parents	2 (6.7)
Other family members	0
Same-sex friends	13 (43.3)
By themselves	8 (26.7)
Age at first injection (years), median (IQR)	23.5 (19–30)
Self-reported drug injection in the last month, n (%)	22 (73.3)
Number of days injected drugs in the last month, median (range)^a^	5 (1, 30)
Number of injections per day in the last month, median (range)^a^	1 (1, 3)

^a^Among those reported drug injections in the last month

Heroin/crack was the most commonly used (last 6 months, 86.7% [*n* = 26]; last month, 80.0% [*n* = 24]) and injected (last 6 months, 96.7% [*n* = 29]; last month, 73.3% [*n* = 22]) drug among the study participants (Table 2). Iranian Crack is a new form of narcotic substance usually containing heroin, acetaminophen, caffeine, morphine, codeine, thebaine and acetylcodeine, so is heroin-based and hence is quite different from common crack cocaine found in the Western countries. Heroin/crack was also the primary drug of choice in the last 6 months (use 76.7% [*n* = 23], injecting 90.0% [*n* = 27]) among participants. Methamphetamine was the second most frequently used drug both in the last 6 months (76.7%, n = 23) and the last month (60.0%, *n* = 18). However, no one reported injecting amphetamine within the last 6 months or the last month. The other commonly used drugs among the participants were methadone (last 6 months, 53.3% [*n* = 16]; last month, 30.0% [*n* = 9]), sedative drugs (last six months, 50.0% [*n* = 15]; last month, 20.0% [*n* = 6]) and codeine (last 6 months, 33.3% [*n* = 10]; last month, 13.3% [*n* = 4]).

**Table 2:** Type of drugs used/injected by participants in focus group discussion of barriers and motivators to participation and retention in a cohort study in Iran, 2016

Type of drugs	Any drug used^**c**^, n (%)		Any drug injected, n (%)		Main drug used/injected in the past 6 months, n (%)	
	In the past 6 months	In the past 1 month	In the past 6 months	In the past 1 month	Used^**c**^	Injected
Heroin/Crack	26 (86.7)	24 (80.0)	29 (96.7)	22 (73.3)	23 (76.7)	27 (90.0)
Methamphetamine	23 (76.7)	18 (60.0)	0	0	2 (6.7)	0
Methadone^a^	16 (53.3)	9 (30.0)	1 (3.3)	0	2 (6.7)	1 (3.3)
Sedative drugs	15 (50.0)	6 (20.0)	0	0	0	0
Codeine	10 (33.3)	4 (13.3)	0	0	0	0
Opium/Opium extracts	8 (26.7)	2 (6.7)	2 (6.7)	2 (6.7)	1 (3.3)	1 (3.3)
Hashish/Marijuana	6 (20.0)	1 (3.3)	0	0	0	0
Nass (powdered tobacco)	6 (20.0)	5 (16.7)	0	0	0	0
Alcohol	3 (10.0)	0	0	0	1 (3.3)	0
Norjizak/Tamjizak^b^	2 (6.7)	2 (6.7)	1 (3.3)	0	1 (3.3)	1 (3.3)
Tramadol	2 (6.7)	2 (6.7)	0	0	0	0
Cocaine	1 (3.3)	0	0	0	0	0
Ritalin	1 (3.3)	1 (3.3)	0	0	0	0
Ecstasy (X)	0	0	0	0	0	0
LSD/Acid	0	0	0	0	0	0
Glue	0	0	0	0	0	0
Others (not specified)	1 (3.3)	0	1 (3.3)	0	0	0

^a^ Non-prescription use

^b^ Norjizak/tamjizak is a narcotic drug mostly used through injection and is produced by a combination of different opioids, steroids, and benzodiazepines

^c^ Including injecting and non-injecting drugs

Table 3 represents self-reported HCV/HIV testing and diagnosis history among participants. A total of 12 participants (40.0%) reported ever being tested for HCV, of whom four (33.3%) reported being HCV positive. No one knew whether they were tested for Anti-HCV antibodies or HCV-RNA. Compared to HCV testing, a larger proportion of participants (63.4%, *n* = 19) reported ever being tested for HIV, of whom most (78.9%, *n* = 15) reported being negative, only person (5.3%) stated that they are HIV-positive and three did not know or report their HIV status (15.8%).

**Table 3:** Self-reported HCV and HIV testing and status among participants in focus group discussion of barriers and motivators of participation and retention in a cohort study in Iran, 2016

Variables	***N*** = 30
Ever tested for HCV	
Yes	12 (40.0)
No	14 (46.7)
Unsure/Did not know	4 (13.3)
Self-reported HCV infection status^a^, n (%)	
Positive	4 (33.3)
Negative	8 (66.7)
Unsure/Did not know	0
Self-reported anti-HCV serostatus^a^, n (%)	
Positive	0
Negative	0
Unsure/Did not know	10 (83.3)
Missing	2 (16.7)
Self-reported HCV RNA status^a^, n (%)	
Positive	0
Negative	0
Unsure/Did not know	10 (83.3)
Missing	2 (16.7)
Ever tested for HIV, n (%)	
Yes	19 (63.4)
No	10 (33.3)
Unsure/Did not know	1 (3.3)
Self-reported HIV serostatus^b^, n (%)	
Positive	1 (5.3)
Negative	15 (78.9)
Did not know	2 (10.5)
Missing	1 (5.3)

^a^ Among those ever tested for HCV in their lifetime

^b^ Among those ever tested for HIV in their lifetime

The goal of this analysis was to provide a description of barriers and motivators to participation and retention in HIV/HCV cohort studies (Supplementary Table 1) as described by the male and female PWID participating in the focus groups. The following section is a summary of the participants’ responses regarding such barriers and motivators:

### Barriers to participation and retention

The majority of participants thought that conducting a cohort study is important and useful and were willing to participate in such study if their major concerns were addressed. The main concerns with participation and retention in such studies were:

#### Breach of confidentiality and misuse of data

Breach of confidentiality was the central core of concerns regarding engaging in cohort studies among PWID, particularly women. A few participants were deeply concerned with misuse of data by police and judicial authorities and getting arrested or incarcerated as a consequence. The fear of being recorded on camera or other devices by research or site staff was cited as an important reason why some might resist getting involved in such studies.

*“ … My family is very well-known. I am an outgoing person myself. My biggest concern is to risk my reputation and the fact that nobody knows that I am injecting drugs. I will get into trouble if my brother or my aunt who is a nurse gets to know this.”* – Male, FGD1.

One female participant joked about the potential consequences of private and confidential information being divulged:

*“…If my family becomes aware of my drug injection, I’ll have to kill myself.”* – Female, FGD2

#### Fear of positive test results

Fear of learning one’s HIV or HCV status was cited as another barrier to participating in cohort studies. Several individuals mentioned that they usually refuse to get tested for HIV or HCV because they believe they cannot face the reality. Some stated that they preferred not to be tested at all because they can assume that they are healthy and continue living with happy thoughts.

#### High level of commitment needed/competing priorities

Some participants believed that getting involved in a cohort study and trying to attend all the potential follow-ups needs a lot of commitment and can be time-consuming.

A number of participants stated that follow-up frequency requirements might interfere with their working schedule and threaten their employment which will eventually lead them to drop out of the study. Similarly, a few participants believed that the time they would spend to commute and attend the visits can be used to look for drugs or find the means to afford drugs. They were also worried that during the study procedures, they may not be able to use or inject drugs and therefore they might experience withdrawal-associated symptoms which are very unpleasant.

#### Marginalization

Homelessness or instability in living arrangements was another barrier drawn from the discussions between participants. Some participants declared that due to their housing situation, they can only be reached by outreach efforts and they have no other means of contact with the outside world. In addition, most stated that they barely have access to transportation facilities from where they live which might make it challenging to participate and particularly retain in the study.

*“ … The place I am hanging out with my friends is a corner in a pistachio garden. One person is injecting drug, the other is preparing drug using a spoon, and the other is using Shisheh (Crystal methamphetamine). Altogether we are about 4. Nearby, another group of 5 are also hanging out. Most are homeless, all they have is in their backpack.”* –Male, FGD3

### Motivators of participation and retention

#### Monetary incentive

Receiving monetary incentive was the top motivator chosen among the study participants to get involved and remain in a cohort study. While the primary incentive was mentioned to be very important to engage people, the majority liked the idea of monetary rewards progressively increasing for each scheduled follow-up as the study progresses, and an additional incentive for successfully recruiting peers and partners in the study.

Along the same lines as money, covering the costs of commuting to study sites or providing transport options for participants was stated to potentially address some of their monetary concerns and can be regarded as a good motivation to keep participants engaged.

#### The thought of a better and healthier life

Most, but not all, participants believed that their participation in such studies can be a solution to their problems or a new hope for them and their families to get better and have a healthier life.

A participant addressed monetary incentive and belief in the possibility of having a better life along with being reassured of data confidentiality as the three keys to success of cohort studies:

*“… In my opinion, three things can make this study successful. First, monetary rewards; so that the study participants do not worry about their time and income anymore. Second, telling participants how this study would make their lives better, easier and safer. Third, building rapport with the participants and reassure them that their information will be kept confidential.”* Male, FGD3

Another participant suggested that such studies can be informative about health and can help them gain a better understanding of their health-risk behaviors:

*“… You can help us increase our health-related knowledge. For example, most men are not aware of methamphetamine use complications. They think that side effects like violence and anxiety are part of their personality and not related to methamphetamines.”* Male, FGD1

#### Protection from police and abstinence camps

Another important motivation for participating and remaining in a cohort study was mentioned to be strategies that can keep people safe from police pursuit and from being sent to drug abstinence camps. A few participants suggested being provided with an introduction letter or a membership card for the study which explains that the person carrying the letter or card is currently involved in a study and therefore should be protected from drug-related police interventions during the study period.

### Barriers to peers and partners recruitment

We asked participants about whether they are willing and able to invite and recruit their peers and partners (i.e. sexual and/or injecting partners) in a cohort study and what they think might be barriers and motivators for themselves in doing so and for their peers or partners to get involved.

Most participants claimed that they know other PWID (between 2 and 8 peers on average) and can invite them to the study. The majority also knew a place or venue where their peers who usually do not visit DICs or utilize services can be approached. Most, but not all, were also eager to invite and bring their sexual and/or injecting partners to the studies with them. However, they felt there is a need to address some problems or barriers first. Apart from fear of data misuse and police arrest that was mentioned by participants to be important barriers for peers and partners to engage in the studies, a few other specific barriers are presented here:

#### People who inject drugs are hard to find

Many participants expressed that injecting is becoming rare among people and therefore it is very hard to find people who inject drugs. They also alluded that majority of PWID do not have a stable location to live and are always moving from one place to another. Many of these people can only be approached in isolated and in many cases remote venues which makes it even more difficult to invite them to the studies.

*“… Nowadays, very few people inject drugs. Of those, not all disclose their injecting status and they mostly can be found in remote venues.” –* Male, FGD1

#### Lack of trust and comfort

While the majority of participants stated that they know someone who is injecting drugs, not all felt comfortable to invite them to participate in the study. Interestingly, some female participants mentioned that female PWID might have less trust in female peers, compared to their male counterparts. So, it would be easier for male PWID to recruit female peers. However, majority stated that they would have no concerns with recruiting their injecting partners to the study, providing that they were interviewed separately. Two female participants said they would feel uncomfortable introducing their partners to others.

*“… I know many people who inject drugs. I am afraid to tell them, but I will do my best. Maybe they are interested, I don’t know.”* Female, FGD2.

### Motivators of peers and partners recruitment

#### Monetary incentives

The same motivators as ones discussed for participation and retention in cohort studies were stated when talking about peer and partner recruitment to these studies. An additional monetary incentive ($2) was stated to be a great drive for participants to invite and engage their peers and partners (i.e. sexual and/or injecting partners) in such studies. Participants also found monetary incentive to be a good motive for their peers or partners to get involved in the study. Alternative incentives such as meals, clothes and methadone coupons were also mentioned to be good incentives for peers and partners.

#### Building trust and positive relationships

Being supportive of people who inject drugs when approaching them was cited to serve as an effective motivator to get them involved in studies. Participants believed that successful engagement of PWID in long-term studies needs a good level of trust and rapport between them and health workers or researchers. It was also alluded to be very important for health workers or researchers to remain kind and encouraging throughout such studies in order to keep people motivated for the whole time.

### Logistics needed for cohort studies

After debriefing the FGD participants about the aims and outcomes of a cohort study, the potential number of visits involved (screening, the one and a half month follow-up and then quarterly visits) and the types of bio-behavioral data begin collected, participants indicated their preferred time, location and other logistics for attending the study visits. The majority agreed on DICs like the one where the FGDs were occurring as their preferred place for the interviews and other study assessments. However, most mentioned they prefer not to be interviewed by the staff at DICs, mostly due to their concerns regarding breach of confidentiality. They were also more willing to be consented and interviewed by same-sex interviewers. For the majority of male participants, the preferred time and days for attending the study visits were between 4:00 and 7:00 pm, any day during the week except Thursdays and Fridays (weekends in Iran calendar). This is while female participants had a wide range of preferred time and days for attending the visits, with half of them agreeing on Tuesdays’ mornings.

While some participants said they are only accessible by outreach workers, some others mentioned they own a phone or have access to a phone and have no problem with giving their details to the study team. Most did not have any objection against using devices like iPad or tablets to collect data during the study visits, and even believed applying such devices is more beneficial since the information collected can be password protected this way.

## Discussion

A comprehensive response to both HIV and HCV among PWID requires robust surveillance of HIV/HCV infections among PWID as well as regular evaluation of harm reduction programs among this population. This study provides a qualitative description of barriers and motivators impacting participation and retention in HIV/HCV cohort studies among PWID in Iran. It is apparent that, despite the seeming willingness of most PWID to get engaged in HIV/HCV cohort studies, there are multiple barriers that affect their participation. In general, a wide range of individual, social and structural factors may affect the decision to engage in cohort studies among PWID.

Similar to what we observed in this study, data confidentiality is often referred to as a great concern in longitudinal studies in which inclusion of personal identifiers (e.g. name and address) is required in order to assure a proper record linkage and an active follow-up [39, 40]. This issue, strongly emphasized by women in our study, can be considered a structural barrier potentially resulting from the existing punitive laws and high levels of stigma and discrimination around injecting drug use in Iran [41]. Although the recent shifts from hard-line drug laws in Iran have succeeded in approaching and engaging PWID in harm reduction services to a large extent [42] and therefore has made them more accessible for research, it appears that further efforts are required to close the trust gap between this marginalized population and the healthcare researchers. Building and maintaining a trusting relationship, reducing stigma through education and awareness programs, using peer workers in research, refraining from collecting personally identifying information as much as possible and securing data gathered in research are a few approaches that help address concerns around confidentiality and security among PWID. Appropriate strategies need to be deployed in order to protect people from getting arrested by police or other undesirable situations related to drugs such as sending PWID to drug abstinence camps.

A further barrier was the fear of testing and therefore engaging in research which seems to stem from perceived inability to deal with a positive test result among PWID or thinking about the detrimental effects of a positive result on their lives. Fear of HIV disease has been showing to contribute as a significant barrier to seeking testing among other vulnerable populations such as men who have sex with men [43]. Additionally, in the context of HCV, fear of being tested has also been represented as a barrier at the stage of decision to get tested for HCV and enter treatment [44]. Anxiety and fear of stigma linked to confirmation of HIV-positive status have previously been represented as persistent issues hindering linkage to care initiatives among at risk populations elsewhere [45]. Providing pre and post-test counselling and educating people about the benefits of testing and potential interventions may help reduce concerns related to HIV/HCV testing. In addition, with the availability of highly effective HCV therapies in Iran, adding appropriate messaging about new HCV medication and the benefits of HCV treatment to individuals as well as communities is necessary and may encourage PWID to seek testing and treatment.

Another two significant barriers to both participation and retention in cohort studies were observed to be the perceived high commitment required and marginalization among PWID.. The first type of concerns can often be mitigated by flexible hours for the study recruitment and assessments and more accessible venues for people to attend the visits. Homelessness was another significant competing priority among PWID. Previous work has shown that homeless people often face numerous complex issues including but not limited to domestic violence, mental illness, and addiction disorders [46–48] and are less likely to be linked to general practitioners or to receive healthcare than the general population [49, 50]. This is while the national evidence suggests that homeless people are at increased risk of HIV and HCV infections [51–53]. Interventions such as task-shifting, mobile outreach, and integrated HIV/HCV and primary healthcare programs as well as housing support might help address homelessness and its attendant heath challenges to a fair extent [45, 54]. While housing support per se is a strong intervention to address many health problems among PWID who are experiencing homelessness, it is likely to also improve the engagement in cohort studies.

Similar to studies in Australia and Canada [55, 56], financial gain was observed to be one of the most important motivations for the majority of participants to engage in studies. However, debates continue on the pros and cons of monetary incentive in addiction research [57]. While some believe that payment is an undue motivation for participation in research among people who use drugs because of their compulsion to buy drugs [58–61], others have argued that reimbursing people for time and inconvenience is a pragmatic way of acknowledging them and has a fair influence on their decisions to engage in research without undermining the voluntariness of such decisions [62, 63]. In addition, it has been indicated that neither the mode (cash vs. gift certificate) nor amount of incentive has a significant impact on rates of new drug use or perceptions of coercion [64]. Financial incentives have been shown to be a strong predictor of willingness to participate in research, increased retention and tracking, and participant satisfaction among PWID [65, 66]. Additionally, evidence suggests that in circumstances where the study population is hard to reach, monetary incentive has the potential to increase participation and decrease sampling bias [57]. Abadie et al. [67] showed that monetary incentive to be the main motivation for PWID to participate in research studies particularly among those who inject frequently or in need of big financial resources. While monetary incentives had a trigger effect to participate in the study, in long term, they were perceived as a reciprocal exchange between PWID and researchers; PWID bring their expertise to the study and so paid for it. Considering participation of marginalized population such as PWID in research as a specialized form of work with proper amount of compensation is critical to build trust with PWID and their communities [68]. Institutional review boards have paid so much attention to “undue inducement” and made researchers to minimize payment levels to research participants to avoid potential undue influence but have little talked about appropriate level of compensation in form of “labor rights and justice”.

The idea of having a better life and getting informed about one’s health was mentioned as two other motivators for our participants to engage in cohort studies. Access to information [69], therapeutic benefits [58] as well as benefits to others [70–72] have been previously cited as potential factors that influence people’s decisions to engage in research. Once again, these findings highlight the importance of education on benefits of HIV/HCV screening, treatment and utilization of existing interventions and services among this atrisk population.

In general, participants were willing to invite and help recruit their peers and partners (i.e. sexual and injecting) in the research studies. While the network of PWID in Kerman has not been fully assessed yet, our FGD findings indicated that PWID were connected to other PWID and can invite them to the study. This is a key factor in using respondent-driven sampling (RDS), a relatively convenient and effective sampling technique that allows collecting representative data from hard-to-reach populations such as PWID through chain referrals [73]. Participants had also a good level of understanding of device-based data collection (i.e. using iPads or tablets) and had no objection against the application of such tools for data collection or storage. Over the past decade, using electronic devices to collect data has become very popular in many epidemiological [74, 75] and clinical studies [76]. Applying electronic devices have been shown to improve the quality of data, make staff training easier and more effective, and reduce the time-lag between data collection, data entry and analysis and use of data [77]. The quality of research studies in Iran can be improved by using such devices and technologies that seems to be acceptable among the key populations at risk.

Besides the concerns regarding data misuse and police interventions which were similar to what has already been discussed, the hidden nature of injecting drug use was addressed as a significant obstacle in the process of peers’ recruitment. Also, in this context, there were some concerns regarding trusting peers and partners. A few motivators including monetary incentives for successful recruitment of peers and partners were suggested to be effective in encouraging people to invite others to research. In addition, creating a positive and supportive environment for people was mentioned to be the key to build trust between PWID and the health sectors and therefore help them easily recruit others to research studies. Establishing and maintaining trust has been shown to be fundamental to success in studies of key marginalized populations [39]. Peer recruitment methods like RDS, strategies to address the needs of population of interest, and the researchers’ developing a good reputation among the studied population have been cited as a few strategies that build trust between researchers and PWID [39].

Our study is subject to several limitations. The participants in the study were a convenience sample recruited through a DIC and from an urban setting in the southeast Iran, therefore findings might not be generalizable to all PWID in the country. However, we tried to enlarge this initial sample through snowball referrals in order to capture as full a range of PWID’s views and discourses that co-exist in this area. Additionally, since data were collected through a focus group process, social desirability bias may affect the findings. Finally, our study only measured the barriers and motivators of intention to engage in HIV/HCV cohort studies among PWID. The real participation rates, barriers of participation, and reasons for lost to follow-up need to be assessed during the implementation of the cohort studies. Despite these limitations, findings from this study are important for better understanding of strategies to promote participation and retention in future cohort studies of PWID Iran.

## Conclusions

This qualitative study provides insights to barriers and motivators to participation and retention in longitudinal research studies among PWID in Iran. Breach of confidentiality and misuse of data, fear of a positive HIV/HCV status, the perceived high commitment required for a cohort study and marginalization are the main barriers to participation and retention in cohort studies among PWID. This is while, addressing issues around data security by strategies to protect people from police interventions, benefits from access to health-related information and services, monetary compensations and building rapports between PWID and researchers were identified as effective motivators to engaging in research. Strategies to reduce stigma associated with injecting drug use, enhance data security, use peer workers more often, provide pre and post-test counselling and education, address the needs of more marginalized groups potentially through task-shifting, mobile outreach, integrated healthcare programs, and housing support are among a few approaches that might help address those barriers and strengthen the motivators.

## Supplementary information

**Additional file 1: Supplementary Table 1.** This supplementary table includes a summary of narratives expressed by male and female participants in the study towards barriers and motivators to participation and retention in a cohort study in Iran. **Supplementary Appendix 1.** This appendix includes the focus group discussion guide for the study.

## Data Availability

The data for this study were generated from the study survey and the transcripts from focus group discussions which are all included in the manuscript.

## Abbreviations

PWID: People who inject drugs
NSP: Needle and syringe program
OST: Opioid substitution therapy
VCTC: Voluntary counselling and testing centres
DIC: Drop-in centre
FGD: Focus group discussion
RDS: Respondent-driven sampling
