# Supplementary material for: Barriers and motivators to participation and retention in HIV/HCV cohort studies among people who inject drugs: a community consultation in Iran
Source: Subst Abuse Treat Prev Policy. 2020 Aug 5;15:56. doi: 10.1186/s13011-020-00298-y (PMC7405425; doi:10.1186/s13011-020-00298-y)
Supplement: Supplementary file 1 — Additional file 1: Supplementary Table 1. This supplementary table includes a summary of narratives expressed by male and female participants in the study towards barriers and motivators to participation and retention in a cohort study in Iran. Supplementary Appendix 1. This appendix includes the focus group discussion guide for the study. [file 13011_2020_298_MOESM1_ESM.docx]

**Supplementary Table 1.** A summary of motivators and barriers to attend and remain in a cohort study on people who inject drugs in Iran.

|  | Motivators | Barriers |
| --- | --- | --- |
| Participation and Retention | - Most participants were interested in primary monetary incentive for enrolling in prevention cohort studies ($3 per hour, [range $3 to $10]). - Majority also liked the idea of monetary incentives progressively increasing for each follow-up by $2 as a motivator for retaining in such studies - Many participants also suggested being reimbursed for the transport costs or transport arrangements being provided for them to commute easily to the place where the study is carried out - Some participants believed that engaging in such studies would help them know better about their health and HIV/HCV status, so it might help them relieve general stress and anxiety - Some participants declared that this study can be a solution or a new hope for them and their family to think about a better and healthier life - Most participants liked this idea that they would be provided a letter or a card that says they are involved in a study and protected from police or camps during the study period | - Most participants were concerned with breach of confidentiality to their friends and particularly families - Some participants were worried that they will be recorded on camera during the study - Some participants were worried about police arrest - Some participants were worried about the test results - Some participants were worried that it may interfere with their work or may not have time to come to the study site - A few participants were worried that they would not be able to use drugs during the time of sessions and that could cause them withdrawal-associated symptoms - Most participants were worried about the transportation facilities to the site - Some participants said that they could only be reached by only outreach team and had no other contacts |
| Recruiting peers and partners | - Most participants knew peers (2 to 8 people) and were able to invite them to the study - Most participants knew a place where they can find peers (2 to 8 people) whom are not attending the DIC routinely, and could invite them. - Most were willing to bring their sexual or injection partners to the sessions with themselves - Most participants interested of secondary ‘recruiting’ monetary incentive if successfully recruited a peer ($2) - Most participants reported monetary incentive, foods, cloths and methadone coupons as good incentive for peers - Some mentioned trust, kindness and encouragement as important motivators to convince people to participate in the study | - The same barriers as reported above for “participation” in the study - Some, but not all, were interested in helping to recruit injecting peers and partners - Most were concerned about being interviewed with their partner - One person claimed that some of their peers are afraid of being arrested and forced to go to abstinence camps |
| Logistics | - Most didn’t have any objection to use devices like iPad or Tablet to collect data - Most preferred an outdoor open environment like a park but only for having the discussions not for giving the tests - Most preferred to be interviewed at the DIC by a same-sex interviewer - Most male participants preferred having the study site working hours in afternoon, between 4:00 and 7:00pm, on any days but not Thursdays or Fridays. Half of female participants preferred visits on Tuesdays’ morning. | - Most participants were concerned about being interviewed by DIC Staff during the visits, due to breach of confidentiality |

**Supplemental appendix 1.** The focus group discussion guide for the study of barriers and motivators to participation and retention in a cohort study among people who inject drugs in Iran, 2016

**Consent Process**

- Copies of informed consent forms should be provided for participants or read for those who cannot read and should be completed in advance. Participants should be provided an opportunity to ask any questions. Group verbal consent should also be audio-recorded prior to each FGD.

**Facilitator’s welcome, introduction and instructions to participants**

**1. Welcoming**

Welcome and thank you for agreeing to take part in this research. You have been asked today to participate in this discussion because your ideas are very important. I realize you are busy, and I appreciate your time and patience.

**2. Introduction**

Introduce yourself and the notetakers/observers, and review the following:

- Who are we and what are we trying to do?
- What will be done with this information?
- Why have we asked you to participate in this research?

**3. About the focus group**

This focus group discussion has been designed to evaluate your current thoughts and feelings about participating and retaining in a long-term cohort study for the purpose of HIV/HCV prevention. The discussion will not take more than one and a half hour.

**4. Privacy and anonymity**

Despite being recorded, I would like to reassure you that the discussion will be anonymous. The recordings will be kept safely in a locked facility until they are transcribed word for word, then they will be erased completely. The transcribed notes of the focus group will contain no identifying information. Therefore, you can try to answer to the questions or comments as truthfully and accurately as possible.

**5. Ground rules**

Make sure the following rules are reviewed for the participants:

- Before we start, I would like to remind you that there are no right or wrong answers in this discussion.
- We are interested in knowing what each of you thinks, so we appreciate if you are honest and share your point of view, regardless of whether you agree or disagree with what you hear. It is very important that we hear everyone’s opinions.
- You probably prefer your comments not to be disclosed to people outside the focus group. Please treat everyone in the group, as you want to be treated by refraining from discussing what you hear today in this group.

**6. Ask the group if there are any questions before we get started and address those questions.**

- Is there any question?
- OK, let’s begin

**7. Turn on the audio recorder**

- May I record the discussion to facilitate its recollection? (if everyone agrees, switch on the recorder)

**8. Warm up**

Go around table:

- Let's start by going around the circle and having people introduce themselves.

**Questions**

Try to ask all the questions below in the order given, however it is more important to maintain the flow of the discussion. Suggested probes have been included. You should try to encourage all participants to engage in the discussion.

**Introductory question:**

1. What do you think about the topic that has brought us here today (prevention studies of HIV and HCV infections in people who inject drugs)?

*Probes for discussion- b*riefly explain:

- HIV and HCV infections and populations at risk
- Ongoing programs to reduce risk of transmission
- The importance of HIV and HCV prevention
- How cohort studies can help health professionals to measure the risk and impact of programs and plan to improve services as needed

**Guiding questions:**

1. If you were asked to participate in a prospective cohort study of HIV and HCV infections and risk behaviors, what kind of concerns might you have?

*Probes for discussion:*

- Health/Safety?
- Confidentiality/Privacy?
- Personal benefits of participating in such study?

1. Can you recognize any potential risks of participating in a cohort study with 1 to 3-month follow-up visits which involve blood sample collections?

a. What would your friends’ or family’s reaction be if they found out you were participating in a research study about HIV and HCV?

*Probes for discussion:*

- Misuse of data?
- Police arrest?
- Interfere with families or other friends, privacy disruption?

1. At every visit a trained interviewer will ask you questions about injection and sexual behaviors. Does it matter;

a. If the interviewer is a woman or man?

b. If the interviewer is a person who is working at the health center/study site or somebody from outside

c. Where the study site is?

*Probes for discussion:*

- Explain the current study site and ask what they like and do not like about it

1. What would prevent you from participating in a long-term study; one that involves multiple follow-up visits, detailed interviews regarding your personal and drug use behaviors, and blood collections?

a. Which of the concerns/risks you mentioned above are most crucial, and would keep you from participating in the long-term study?

1. If we were able to address the above concerns/risks, would you be willing to participate in a long-term study with one baseline and multiple follow-up visits?

*Probe for discussion:*

- If the answer is No, ask about the reasons

1. We are interested in recruiting people who inject drugs who are 18 years or older and living in Kerman and are willing to participate in our study;

a. Do you know anybody eligible for this study? If so, how many?

b. Would you be willing to tell them about this research study?

*Probe for discussion:*

- If the answer is No, ask about the reasons

1. Some injecting drug users are hard to reach; they rarely visit DICs, they are not accessible by outreach team and cannot be found at street locations/venues;

A. Do you know where we might find these people who would be eligible for this study?

b. How would you suggest we find these people who might be eligible for this research study?

c. What does motivate them to participate in the study?

d. Would you be willing to tell them about this research study?

*Probe for discussion:*

- If the answer is No, ask about the reasons

1. This small-scale study requires that you attend baseline visit, one and half-month and three-month follow-ups;

a. How do you feel about this?

b. What issues or concerns do you think might come up regarding these frequent follow-up visits?

c. Do you and your peers have cell phones; do you and your peers have reliable phone contacts, an email or a permanent address that we can use to contact you for reminders?

d. Would you be comfortable giving research staff the names and contact information (phone, email, Facebook, etc.) of friends and family for us to locate you and remind you for follow-up visits?

e. What days (from Saturday to Thursday) and times (morning, afternoon or evening hours) are you and your peers most likely to return for follow-up visits?

1. How would you suggest we ensure that research participants return for follow-up visits?

*Probe for discussion:*

- Explain follow-up visits are perhaps just as, if not more important than, baseline visits

1. What form of compensations would be more appreciated by study participants?

*Probe for discussion:*

- Ask about the type of incentives? if cash, how much?
- Overall transportation costs to come to the study site?

1. In the future, we might decide to follow participants beyond the 3 months follow-up. It would be the same as this study, but participants will be followed for a longer time and vising us at the study site for every three months.

a. How would you feel about participating in a longer study e.g. 2 year?

b. Does it affect your willingness or interest to participate in the study?

c. What can be done to motivate people to engage and retain in such long-term follow-up study?

1. One thing we are considering adding to the next phase of the study is to ask participants to bring their injecting or sexual partner(s) into the study;

a. How would you feel about this?

b. What potential concerns might you have with this partner study?

1. Instead of paper-based questionnaire, we are thinking of using iPads to collect data. These devices are programed to collect and store data accurately and securely;

a. What potential concerns might you have with using such technology to collect data during the interview sessions?

**Conclusion**

- Let’s summarize some of the key points from our discussion. Is there anything else?
- Do you have any questions?
- Thank you for taking the time to talk with us. This has been a very successful discussion
- Your opinions will be an asset to the study. We hope you have found the discussion interesting

Please, write your report based on the results of the focus group. Please remember to maintain confidentiality of the participating individuals by not disclosing their names.
